# Deletion variants calling in third-generation sequencing data based on a dual-attention mechanism

**DOI:** 10.1093/bib/bbae269

**Published:** 2024-06-08

**Authors:** Han Wang, Chang Li, Xinyu Yu, Jingyang Gao

**Affiliations:** College of Information Science and Technology, Beijing University of Chemical Technology, North Third Ring Road 15, 100029, Beijing, China; College of Information Science and Technology, Beijing University of Chemical Technology, North Third Ring Road 15, 100029, Beijing, China; College of Information Science and Technology, Beijing University of Chemical Technology, North Third Ring Road 15, 100029, Beijing, China; College of Information Science and Technology, Beijing University of Chemical Technology, North Third Ring Road 15, 100029, Beijing, China

**Keywords:** structural variant detection, dual-attention mechanism, third-generation sequencing

## Abstract

Deletion is a crucial type of genomic structural variation and is associated with numerous genetic diseases. The advent of third-generation sequencing technology has facilitated the analysis of complex genomic structures and the elucidation of the mechanisms underlying phenotypic changes and disease onset due to genomic variants. Importantly, it has introduced innovative perspectives for deletion variants calling. Here we propose a method named Dual Attention Structural Variation (DASV) to analyze deletion structural variations in sequencing data. DASV converts gene alignment information into images and integrates them with genomic sequencing data through a dual attention mechanism. Subsequently, it employs a multi-scale network to precisely identify deletion regions. Compared with four widely used genome structural variation calling tools: cuteSV, SVIM, Sniffles and PBSV, the results demonstrate that DASV consistently achieves a balance between precision and recall, enhancing the F1 score across various datasets. The source code is available at https://github.com/deconvolution-w/DASV.

## Introduction

Genomic structural variation (SV) refers to changes in base sequences and positional relationships in genomes with a length greater than 50bp [1]. SVs that affect gene expression in numerous ways can affect biological traits that lead to genetic diseases. The large deletion of gene segments stands as a primary causative factor for many such diseases. For instance, Duchenne muscular dystrophy is a serious neuromuscular genetic disorder in which deletion accounts for 55–65% of the total types of genetic variations [2], and a large gene deletion in $\alpha $-globin causes $\alpha $-thalassemia, which comprises a group of disorders due to abnormal hemoglobin synthesis[3]. Spinal muscular atrophy is a common autosomal recessive genetic disorder that is also caused by a gene segment deletion[4]. Therefore, the accurate detection of gene deletions is essential for the diagnosis of such genetic diseases.

Currently, SV calling technology based on high-throughput sequencing data is developing rapidly; various algorithms have been generated and genome-wide variant calling has been applied to multiple species. The contributions of various algorithms, such as ReadDepth based on sequencing fragment depth [5], BreakDancer based on paired-end mapping (PEM) [6], Socrates based on split read (SR) method [7], TIGRA based on *de novo* assembly [8], have made to SV calling detectable in high-throughput sequencing data.

However, the NGS represented by Illumina HiSeq2500 has issues such as short read length (only 250–300bp) and susceptibility to base pair mismatch. To obtain accurate and long splicing results, it is necessary to have a high coverage of sequencing, which leads to more errors and increased costs. The above factors will all affect the accuracy of downstream analysis, including SV calling. Based on the advantages of long reads, balanced sequencing coverage and the absence of systemic bias in sequencing, single-molecule sequencing can cover a wide range of genomes. Therefore, it is suitable for detecting longer deletions in genomic segments.

Previous work in the field of SVs detection has seen the emergence of several tools aimed at improving the accuracy and completeness of SVs analysis. Tools such as Sniffles[9], SVIM[10], cuteSV[11] and PBSV[12] used traditional methods to detect SVs in nanopore sequencing data. However, they were often impacted by factors such as the alignment method, data quality and, particularly, coverage depth. There have also been attempts to use neural networks in SV detection with good results. Zheng [13] and Ryan [14] both used deep convolutional neural networks to improve SVs detection, highlighting the benefits of using more automated and generalizable techniques for variant calling. However, gene sequences are inherently closer to human language than to images; we considered incorporating a similar approach to language modeling in our model and employ an attention mechanism to enhance the response to deletion regions.

Therefore, considering the long reads advantages of third-generation sequencing data, we propose a new method named dual attention structural variations (DASV), which uses feature fusion for deletion variants calling based on read alignment. The DASV algorithm incorporates the following main steps:

(1) Convert gene alignment information into images. This would map the deletion variants features of genes to the corresponding regions of the image, which is beneficial for the local perception of data features.
(2) Combine images with genomic sequencing data. The dual attention mechanism would provide the model with the ability to understand data from multiple modalities, combining the advantages of images and sequences to enhance the effectiveness of structural variant calling.
(3) Use a multi-scale network to learn features. The backbone network uses Resnet, where residual connections enhance the model’s expression ability and facilitate structural variant calling. Therefore, considering the long reads advantages of third-generation sequencing data, we propose a new method named DASV, which uses feature fusion for deletion variants calling based on read alignment.

To verify its effectiveness, we compared DASV with the advanced third-generation variation detection tools, SVIM, Sniffles, PBSV and cuteSV. The results showed that DASV has notable advantages for data with high coverage depth, and obtains a high recall rate and F1 score.

## Methods

The DASV method rapidly detects genomic deletions by converting gene alignment information into images and using a dual attention mechanism to combine the image information with genome sequencing data. Whole-genome sequencing data are often immense, and variants are often relatively difficult to directly detect by comparing individual and reference genomes. This method innovatively converts alignment information into images, combines it with textual information about genes and applies a multi-scale network to rapidly detect deletion variants. We designed DASV based on four concepts as follows:

(1) sequence alignment map (SAM) files are filtered to ensure the quality of the comparison;
(2) the generated loci of images are counted according to the deletion range of SAM files to generate images of high-frequency regions;
(3) deletions are detected using a multi-scale network;
(4) the results of multiple tools are integrated with the combiSV algorithm to form a relatively standard benchmark set[15].

**Figure 1:**
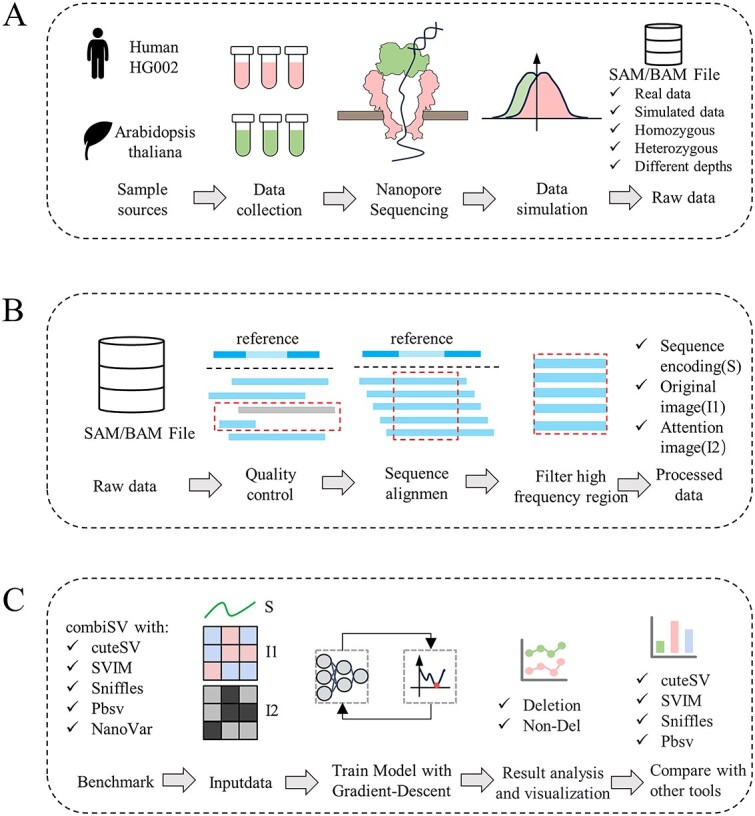
Workflow of our method. A describes that we collected sequencing data from Human and Arabidopsis thaliana and conducted simulations. In the end, we obtained real sequencing data and simulation data (including heterozygotes and homozygotes) with different depths. B describes that after obtaining the raw data, we conducted quality control, sequence alignment and filtered high-frequency region. Then we generated sequence encoding(S), original image(I1) and attention image(I2) based on the high-frequency region. C describes that we used combiSV to create a benchmark and trained a neural network. Finally, we compared and analyzed the results with the other four tools, and visualized them.

Then we compared the precision, recall and F1 score between DASV and four other mainstream tools under the benchmark. The workflow is shown in Fig. 1.

### Pre-processing gene files

#### Quality control of gene files

The SAM file contained sequenced fragments with low alignment quality that would affect downstream analyses and detected variations, thereby resulting in an increased false positive rate. Therefore, such fragments had to be filtered out. Sequenced fragments with an alignment quality number <20 were removed, as were those with a FLAG identifier of 4 and >256. This ensured that reads not comparable with the reference sequence and those failing quality control were eliminated. The false positive rate of the results was reduced using quality-controlled data for variant calling.

#### Framing the range of variation

Deletion features were obtained based on the CIGAR field of the quality-controlled SAM file. The deletion ranges on each read were counted, and variation ranges within the same region were merged. The number of deletions within the same region was counted, and high-frequency regions were selected according to the average sequencing depth of the file to generate an image of the high-frequency region.

#### Sequence encoding

Base sequence of a sample genome was encoded within the framed variation range using the pre-processed BAM file. Using the traditional hard coding method, alignment results of deletion loci were encoded as 9, and information about A, C, T and G bases were encoded as 2, 3, 4 and 5, respectively. And data whose length is shorter than the specified threshold (100) will be padded with zeros(0) up to 100, thereby normalizing the data size for parallel training. The amount of data in the gene file itself is immense, and one-hot encoding would increase the feature space and lead to extremely slow encoding speed. The advantage of using hard coding was that direct mapping of features avoided dimensionality issues and improved calling efficiency.

#### Image generation of high-frequency regions

Analysis of real data revealed that the length of most deletion variations was hundreds to thousands of bp, causing difficulties with completely representing an entire deletion region by generating one image per region. Therefore, to generate the image in the high-frequency region, the sliding window was set to 100 and the stride length was set to 50. Canvas length and width were set to 100 pixels, with each pixel row representing a read, and all reads were arranged from top to bottom and left to right. Images that were <100 pixels were padded with whitespace and those that were >100 pixels were compressed so that the final image size was 100*100 pixels.

**Figure 2:**
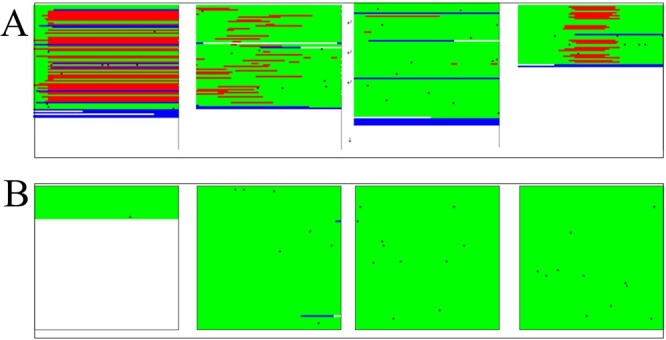
Original image(I1). Mapping generated images of different genes fragment. We randomly selected four instances. The red color represents the deletion regions. A shows data with deletion variants. B shows data without deletion variants.

The overall background of the images was white, which means the three RGB channel values were all 255. The letters D, M, S and I in the CIGAR fields represent deletion, matched alignment, soft cut and insertion, respectively. To generate images, these modes, respectively, corresponded to four RGB values (255, 0, 0),(0, 255, 0),(0, 0, 255) and(0, 0, 0), and the different CIGAR fields were red, green, blue and black, respectively. Fig. 2 displays examples of image data: Panel A includes four instances with deletion variant, Panel B includes four instances without deletion variants. The sequence information in BAM/SAM files is converted into images, enabling the linkage of different sequences to express more dimensional information. The horizontal and vertical coordinates of each image represent locus information and coverage depth, respectively, and the color reflects comparisons between sample and reference genes. This connects the features of deletion variants from isolated read to the regional features and greatly facilitates the deletion variants calling in downstream tasks.

### Multi-scale network

#### High-level approach

**Figure 3:**
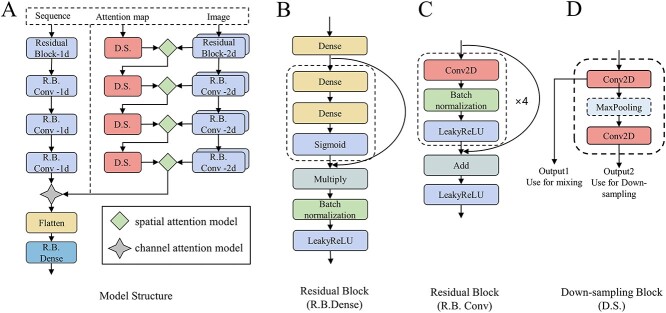
Model structure and some modules. A is the model structure, and spatial attention model and channel attention model are from Fig. 5. The model has three inputs, understood from different perspectives. B is the residual connection used in the dense layer during the final down sampling. The original matrix is recalculated through multiply operation and residual connection. C is the residual connection used in the Conv(both 1d and 2d) layer during the backbone; retains important features of the original matrix through add operation. D is the down sampling module of the (I2), output1 is used for feature fusion and output2 continues with the next round of down sampling. The maxpooling module is represented by a dashed box because the first layer was not down sampled.

The DASV method could detect deletions in long-read sequencing data. It converts sequence alignment information into images and encodes them using integers. A channel attention mechanism is used to fuse images mapped by the CIGAR field in the SAM/BAM file with the encoded data to allow for communication and interaction between the two channels, which enables the model to learn the correlation of different channels. Correlation is learned by assigning weights to the features of each channel. Fig. 3(A) shows that the backbone network uses the ResNet, which is easy to optimize and can gain accuracy from considerably increased depth.

In our method, the spatial attention matrix is added to the image mapped using the CIGAR field to increase the proportion of the D field. Moreover, the base sequence of the sample encoded genome used the image as the feature weight of the sequence data, which is multiplied (a matrix multiplicative weighting method) by the channel width of the sequence sample to achieve the rapid detection of deletions. The DASV retains complete sequence information. Meanwhile, it extracts deletion features for rapid positioning and calling. This method also is robust for sequencing data of different depths, which means an excellent calling ability of SV in sequencing data with high and low depths of coverage.

#### Spatial attention method of the image

**Figure 4:**
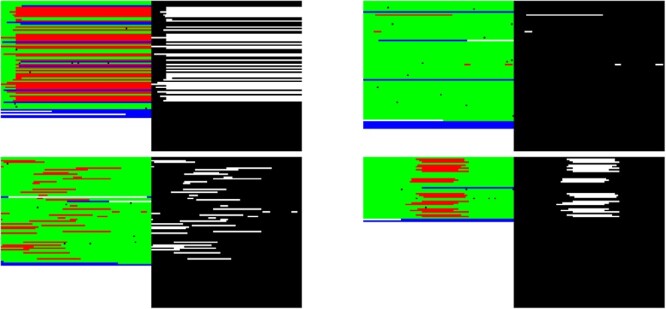
Attention image(I2). Set the red regions in the generated image to white and the rest to black to enhance the model’s response to deleted regions.

**Figure 5:**
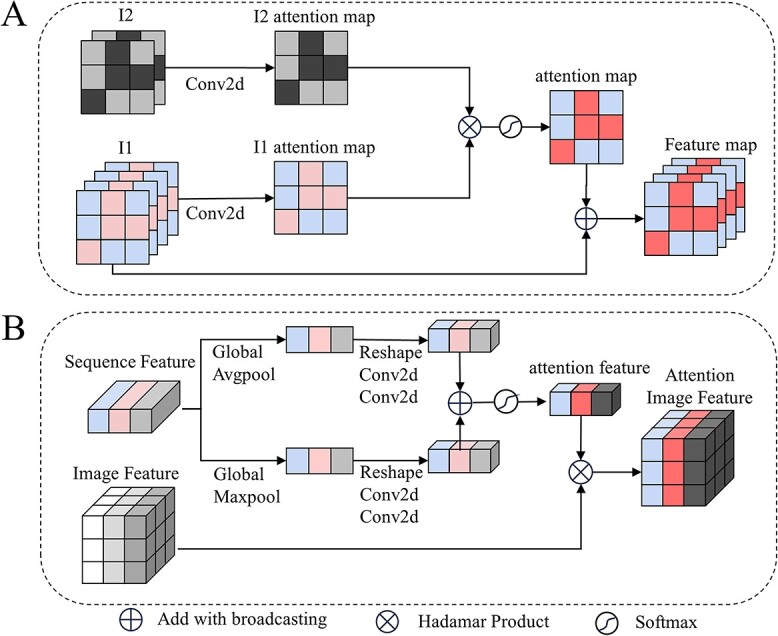
Attention mechanism structure. A is the spatial attention mechanism that selectively focuses on relevant regions. B is the channel attention mechanism, which determines the importance of each channel in feature maps.

DASV applies the spatial attention model to focus the features on the image to the regions of deletion and enhance the meaningful feature by weighting to enhance the importance of the D region of the CIGAR field. In Fig. 4, original images(I1) are converted from the four matching modes of the CIGAR field, which are located in the left columns. The columns on the right are attention images(I2). The corresponding RGB value is (255, 255, 255) when the deletion part of the CIGAR field is mapped to the image, while the remaining part is (0, 0, 0). Convolution operations are performed on the original image(I1) and the attention image(I2) to enable the network to automatically learn their features. It can be seen as a data augmentation operation. After the convolution, the I1 and I2 are multiplied to obtain an attention map with enhanced deletion regions. To avoid gradient explosion or vanishing, the softmax function is used to normalize the attention map and limit the values of the attention map to 0-1. Finally, the attention map is multiplied by the original matrix to obtain the final output. Fig. 5(A) is a schematic diagram of feature extraction based on the spatial attention model.

#### Sequence and image fusion

Common fusion methods include addition and concatenation operations. The former mathematically sums pixels corresponding to the features. The size of feature maps and the number of channels are the same, but the information on each site will change. The latter concatenates the features together. The size of the feature map remains the same but the number of channels increased. Both methods do not consider the effects of different features’ weights on model performance. The feature fusion method designed in this study is based on the attention mechanism of the channel domain, which weights the attention of different channel features. Encoded images serve as the channel attention of the sequence and weight is applied to each channel feature map of the sequence to represent the correlation of that channel with the deletion feature. More weight represents closer correlations with the variation. In Fig. 5(B), the input feature map is generated using the spatial attention module. It is downsampled using Global Average Pooling(GAP) and Global Max Pooling(GMP) to obtain its global receptive field. After GAP and GMP calculations fix the weight vectors, convolutional layers are used to recalculate them, thereby enhancing learnability. Following the summation of the two sets of weight vectors, they are normalized using the sigmoid function. Finally, the sequence (S) is multiplied by each channel, facilitating the recalculation of the importance of the original sequence features.

### Acquisition and evaluation metrics of standard benchmark sets

The acquisition of the benchmark set is divided into two parts. The first part experimental data, HG002, used the new benchmark developed by NIST for large insertion and deletion variants in 2020 [16]. The NIST synthesized 19 analytical methods to develop a new SV benchmark set for the Ashkenazi Jewish trio HG002 sample from the Personal Genome Project, which is available as NIST Reference Material 8392. The benchmark set contains 12 745 independent variants >50 bp, including 7281 insertion callings and 5464 deletion callings. The other part of the benchmark set was obtained using the combiSV method from another real dataset [17], and the results from five structural variant detection methods were combined to create a detection set with higher recall and precision.

Using the combiSV method we evaluated the performance of seven SV detection algorithms for long-read data with 24 600 structural variants (SV events). The results of seven SV callers were analyzed using the Nanopore, PacBio and PacBio HIFI data, and a benchmark set was finally generated by integrating the results using cuteSV, SVIM, Sniffles, pbsv and NanoVar tools. The balance between recall and precision determined using cuteSV or SVIM was good for most datasets and yielded the best overall performance. The precision of cuteSV was the highest for all datasets, but its performance was lower in the PacBio HiFi dataset with coverage <30x. The PBSV demonstrated the highest accuracy, benefiting from its specific design for PacBio systems. Both nanoSV and NanoVar had high recall but at the cost of high false positive rates. Therefore, we applied combiSV to integrate various tools to obtain a more accurate benchmark. Additionally, we considered the recall and precision of experimental detection using F1 score (Detailed in supplementary’s Evaluation metrics), where a higher F1 score indicates a better result.

## Experimental results

We constructed the DASV method that can identify genomic deletions. We compared the performance of DASV with the following popular SVs calling tools: cuteSV, SVIM, Sniffles and PBSV to validate the performance of DASV using simulated and real datasets.

### Dataset

The samples of the F1 hybrid of Arabidopsis thaliana[18] and samples of the GIAB BioProject PRJNA200694 [19, 20] were selected to construct a real sample set, detail information can be found in Table 1. Raw sequence data about the Ashkenazim Trio (including HG002, HG003 and HG004) and the aligned files were obtained from GIAB (https://ftp.ncbi.nih.gov /giab/ftp/data/AshkenazimTrio/). Additionally to the experimental data, eight PacBio SMRT sequencing fragment datasets were simulated for homozygous and heterozygous variations to compare the ability of the methods to detect variations with different lengths of deletions and sequencing depth. Sim_A90X,65X,45X,20X and Sim_B90X,65X,45X,20X were simulated datasets of homozygous and heterozygous variant groups, respectively, to analyze the ability of DASV to detect genomic deletions.

**Table 1:** Experimental samples

Sequencing technology	Sample	Sequencing depth	Average length of sequenced fragments (bp)
CLR	Arabidopsis thaliana F1 progeny	155	11 260
CLR	GiaB HG002 (son)	69	8540
CLR	GiaB HG003 (Father)	32	6284
CLR	GiaB HG004 (Mother)	30	7286
CLR	HG002	28	13 478

### Comparing the results of whether to perform feature fusion

**Figure 6:**
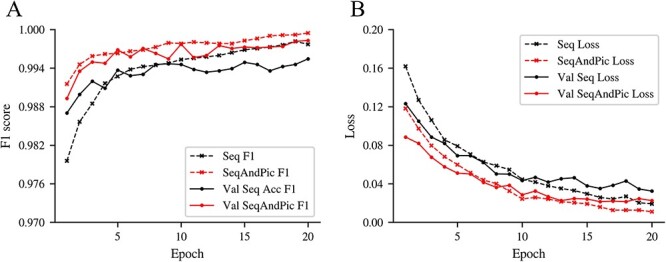
Comparison of F1 score and loss. The results show that after using the method proposed in this study to convert the sequence into images and fuse them, the loss is reduced, and the F1 score is considerably improved.

**Figure 7:**
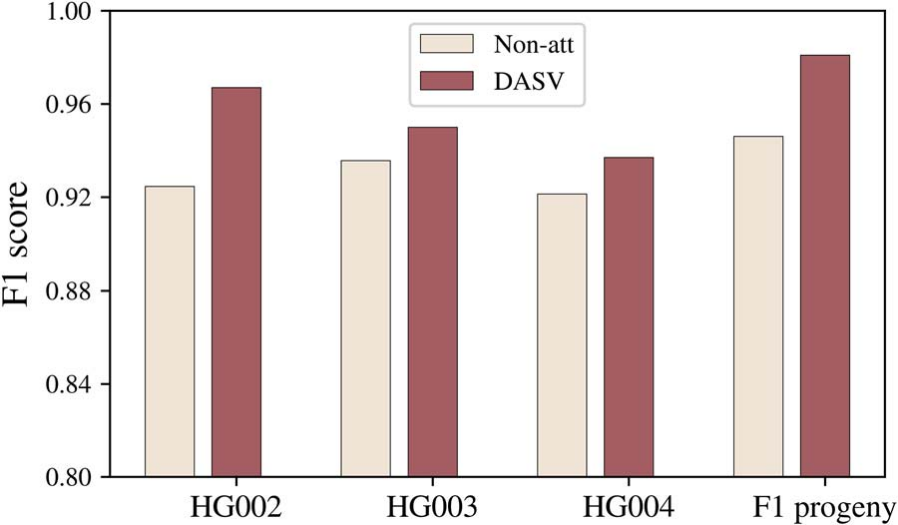
Experimental results of different experimental data(F1 score). There have been notable improvements in both HG002, HG003, HG004 of the GIAB BioProject PRJNA200694 and F1 progeny of Arabidopsis thaliana. The Not-att in the legend represents that we only use a simple fusion of the features. The DASV is the dual attention mechanism.

We have compared the results of only extracting sequence information and fusing sequence and image features to verify the improvement of the dual attention mechanism in detection performance. Using the dual attention mechanism to integrate images and sequences amplified the features of deletion variants, making the network learning process more focused on deletion variants and the training process more stable in convergence. Fig. 6 shows the accuracy and loss comparison of whether to perform feature fusion. As shown in Fig. 7, there are clear differences in the results of different methods across various real datasets. It is evident that the F1 score has been considerably improved following feature fusion. Detailed data are available on Supplementary Table 3.

### Experimental results of real data

#### Analyses of Ashkenazi Jewish trio HG002 samples on the standard set

**Figure 8:**
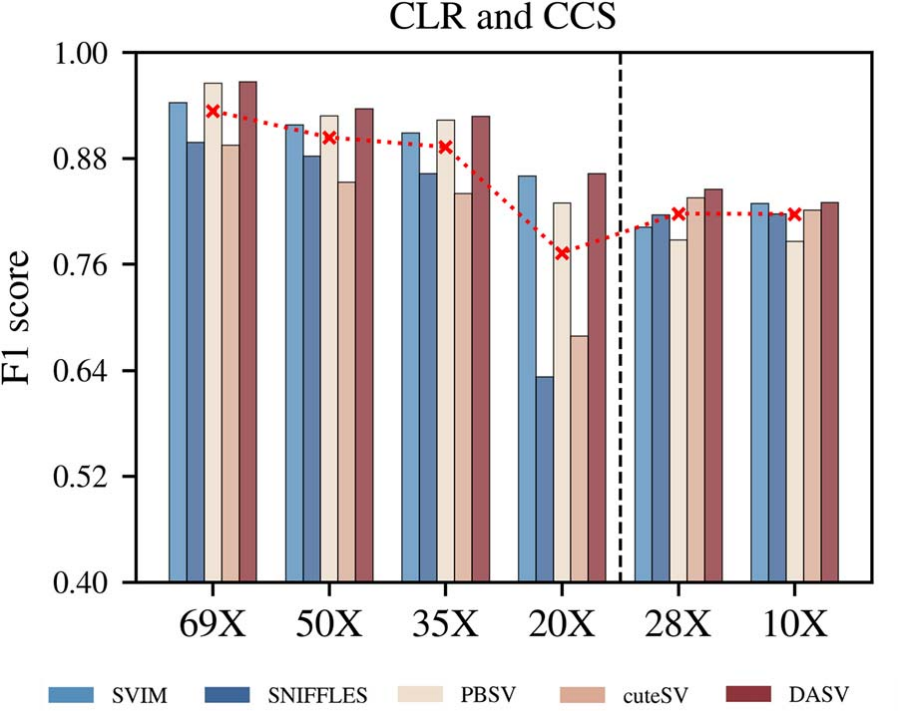
Performance of different tools for different coverage depths on the HG002 sample. We analyses different method’s data: CCS and CLR. The results show that both overall and partially, the F1 score decreases as the coverage depth decreases.

The valid dataset was HG002, and the standard set was Reference Material 8392 Human DNA, which includes 5464 deletion callings. The results were compared with the standard set to quantify the ability of various methods to detect deletions. We analyzed deletions in samples using SVIM, Sniffles, PBSV and cuteSV, using precision, recall and F1 score of the calling results as comparison metrics. On the bar plot, Fig. 8, the results on the left side of the black dashed line were obtained from the continuous (noisy) long reads (CLR), and the results on the right side of the line were obtained from circular consensus sequencing (CCS). It could be clearly seen from the red dashed line that the coverage depth will notably affect the detection results. The F1 score decreased as the coverage depth decreased. In all experiments, the F1 score of DASV was the highest. Detailed data are available on Supplementary Table 4, the precision of DASV was lower than the Sniffles and PBSV, but the recall and F1 scores were higher than other tools. Overall, DASV achieved the best results.

#### Family sample analysis

Here, we calculated F1 score for the F1 hybrid of Arabidopsis thaliana and HG003 and HG004 of GIAB and used the benchmark set with combiSV, which synthesized the results using the cuteSV, SVIM, Sniffles and pbsv tools.

**Figure 9:**
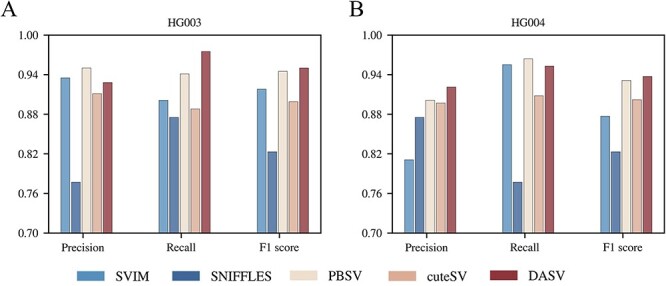
Performance of different tools on HG003 and HG004 samples.Both Precision and Recall are important evaluation metrics. During the experiment, we found that the Precision of PBSV was higher than that of DASV, but the Recall of DASV was higher than that of PBSV. Therefore, we used F1 score to comprehensively evaluate different methods. Obviously, both on HG003 and HG004, DASV outperforms PBSV.

**Figure 10:**
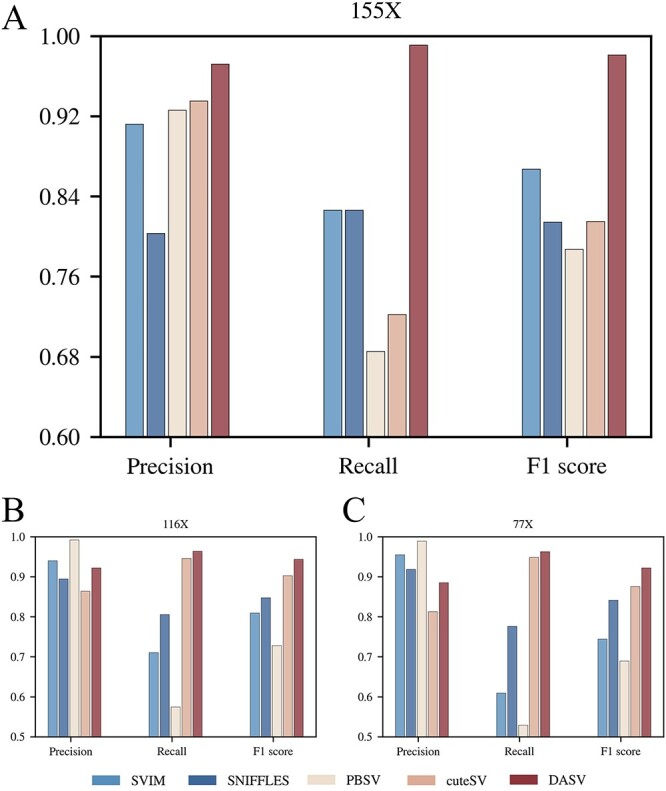
Performance of different tools for different coverage depths of Arabidopsis F1 progeny samples. At coverage depths of 116X(Fig. 10.B) and 77X(Fig. 10.C), DASV performed similarly to human data. But when the depth reached 155X(Fig. 10.A), DASV achieved maximum values on all three metrics.

Fig. 9 and 10 showed the results of the HG003 and HG004 of GIAB samples and the F1 hybrid of the Arabidopsis thaliana sample, respectively. Detailed data are available in Supplementary Tables 5 and 6. The DASV method had higher F1 score and recall value than the other tools, as shown on Fig. 9. This indicated that DASV’s results in HG002 were not accidental and can work well on commonly used datasets. Especially in Fig. 10(A), DASV demonstrated better precision than PBSV, achieving the best results. Although the detection performance of DASV and the other four tools decreased as coverage depth decreased, the decline was more pronounced for the other four tools, especially PBSV. DASV mitigated this performance degradation and maintained better precision and recall.

### Results from simulated data

To further comprehensively investigate performance of the DASV method, we additionally analyzed results from simulated data sets. The simulated data could not fully reflect the genetic data characteristics of individual organisms, but their advantage is controllability, which can effectively test the performance of tools.

We simulated a specific number of variant genes using a parameter file generated by SURVIVOR[21] to set the type, quantity and length of simulated variants. The mode of sequences was learned using the simulated sequencing tool PaSS[9] to obtain the original FASTQ file containing the gene sequencing data. The FASTQ file was then compared with the reference genome using the NGMLR[21] tool to obtain the BAM file.

**Figure 11:**
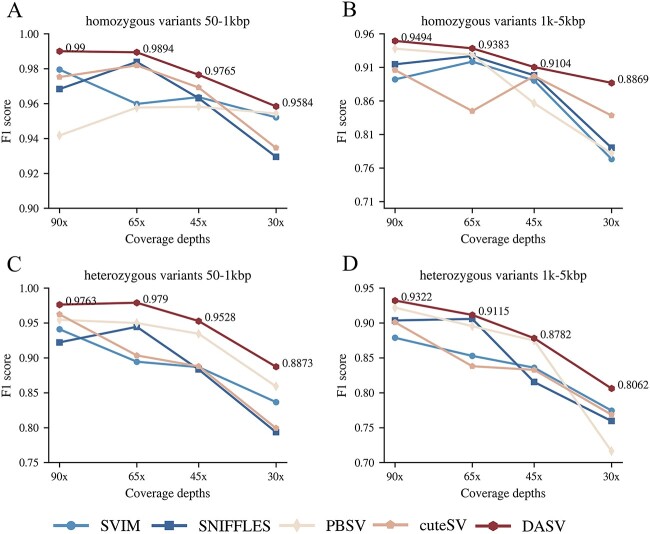
The changing trend of F1 score. The DASV method based on data with different coverage depths and deletion lengths achieved a higher F1-score than all other tested tools and performed better at higher coverage depths.

The simulation experiments included homozygous deletion of Sim_A{90X,65X,45X,20X} and heterozygous deletion of Sim_B{90X,65X,45X,20X}, respectively. The generation method for both datasets was the same. Each set of experimental data contained different lengths of deletions and coverage depths. Simulated sequences’ coverage depths of 90x, 65x, 45x and 20x were generated for deletions between 50 and 1000 bp and between 1000 and 5000 bp, respectively. The average read length was 20k bp, and the average calling error rate was 15%. Supplementary Tables 7(a&b) and 8(a&b) show the experimental results of the homozygous and heterozygous deletions, respectively. The results of the simulated homozygous deletion of Sim_A{90X,65X,45X,20X} and homozygous deletion of Sim_A{90X,65X,45X,20X} revealed that heterozygous variants were more difficult to detect. The recall and F1 score were the best under high coverage depth compared with the other four tools, and the precision performance was also quite outstanding. But for large deletion variants calling, the recall of the other four tools has decreased. In contrast, DASV maintained a high recall. Even with low coverage depth data, there can be a recall of 82.16%. Fig. 11 shows that the DASV method based on data with different coverage depths and deletion lengths achieved a higher F1 score than all other tested tools and performed better at higher coverage depths.

## Discussion

Third-Generation Sequencing Technology has revolutionized genomic studies, enabling single-molecule sequencing of DNA fragments without PCR amplification. And its distinct feature of long reads considerably reduces the subsequent workload of Genome Assembly and Annotation, leading to substantial time savings. However, it also exhibits a higher error rate, which necessitates compensatory measures such as increased sequencing depth. Improvements in computational power and deep learning have greatly advanced computer science. These advancements have enabled a more in-depth analysis of data that surpasses human comprehension and have transformed the way we address complex scientific challenges. Moreover, deep learning algorithms have enabled a more effective analysis of Third-Generation Sequencing data, thereby enhancing the accuracy and efficiency of genomic analysis. This development provides powerful new perspectives and tools for unraveling the mysteries of life.

In this study, we conducted a preliminary analysis of third-generation sequencing data and introduced a dual attention mechanism for calling deletion variants. Following comprehensive data analysis and quality control procedures, we employed a predetermined set of rules to generate tri-channel images from the sequencing data. These images were then integrated with the original sequences, facilitating rapid and precise identification of deletion variants. To underscore the performance of DASV, we conducted validations with both real and simulated datasets. The results demonstrate that DASV surpasses all currently prevalent tools, particularly in terms of F1 score. Furthermore, given the high error rates associated with third-generation sequencing, we assessed the performance as sequencing depth decreased. The findings revealed that our proposed method, DASV, consistently delivered impressive results across various sequencing depths. We attribute the effectiveness of our study in calling deletion variants to two key factors:

First, the conversion of sequence data into image data and their integration with the original sequence data. Image data, often more intuitive, possess translational invariance during computation, thus maximizing the preservation of features related to deletion variants. Sequence data provide the model with raw information, mitigating potential information loss during conversion into images (primarily due to the use of 100-sized sliding windows).

Second, the residual connections in ResNet preserve essential features to the maximum extent. ResNet addresses the problem of vanishing gradients that arise with increasing model depth. Residual connections, which are parameter-free, retain the original information related to deletion variants, thereby enhancing the smoothness and stability of network training and improving convergence capability. Compared with traditional variant calling tools such as cuteSV, SVIM, Sniffles and PBSV, this deep learning-based method extracts biological features of sequencing data from vast datasets. The multimodal learning capabilities of deep learning enable the conversion of sequence data into image data, contrasting with traditional methods that are limited to processing only one modality. We established three sets of control experiments: long-read versus short-read data, real data versus simulated data and homozygous variants versus heterozygous variants data. The methods presented in this study consistently achieved notable results across all contexts. Traditional methods encompass a plethora of sequencing data processing techniques that merit consideration. They feature rigorous inferential procedures, a quality lacking in deep learning. In future work, our primary focus will be on:

**Comprehensiveness of variant calling.** This study currently focuses exclusively on experiments related to deletion variants calling. We intend to broaden our research to include other mutation types, such as insertions and base substitutions, to achieve excellent outcomes across various downstream applications. Ultimately, we aim to further develop and package this technology into user-friendly software.

**Advancement of the model.** The rapidly evolving landscape of deep learning technologies continues to drive our exploration of novel applications and innovations in the field of variant calling, and we have initiated related work. We anticipate notable advancements in the near future, particularly in exploring models such as Transformer.

**Extrapolation capability of our method.** Given a sufficient volume of data, deep learning has demonstrated its capacity for extrapolation. We aspire to collect an extensive array of datasets, gathering information from a diverse range of biological sources, thereby transcending the limitations of training data and enhancing the generalization capabilities of deep learning. We aim to deploy this technology across multiple domains, evolving it into a comprehensive large-scale variant calling model.

## Conclusions

Understanding the large variation of DNA sequences in the genome can provide a basis for the diagnosis and treatment of diseases. In this study, we present a method for deletion variants calling based on a novel dual-attention mechanism, which is named DASV. Our method considers the long-read advantages of third-generation sequencing data, innovatively converts genetic information into images, enhances local response to deletion regions and then uses the attention mechanism to integrate the images with original information. The attention map extracted from the image more effectively highlights deletion loci, improving the sensitivity and robustness of detection. To verify the performance of DASV, we conducted multiple experiments on real data and simulation data. The results revealed that DASV has improved detection performance compared with existing deletion variant calling tools, achieving higher detection precision while ensuring the recall, which means a higher F1 score. This will contribute to a more comprehensive exploration of deletion variants in the biological genome and provide new solutions for genomic science research.

**Key Points**

- A deep learning tool capable of detecting deletion variations on third-generation sequencing.
- Designed a method to understand data from both sequence and image perspectives.
- Enhancing the response of models to deletion variations using dual attention mechanisms.
- Comparing the performance of the state-of-the-art tools with the proposed model, DASV achieved the best results.

## Supplementary Material

supplementary_bbae269

supplementary-table_bbae269

## Data Availability

All the real datasets used in this study are publicly available and accessible. Detailed data sources can download from GIAB (https://ftp.ncbi.nih.gov/giab/ftp/data/AshkenazimTrio/). And simulation data are upload to https://github.com/deconvolution-w/DASV.

## Author Biographies

**Han Wang** is a master's student at Beijing University of Chemical Technology. His research interests are AI for biomedical including medical image processing, biological sequence analysis, etc.

**Li Chang** is a master's student at Beijing University of Chemical Technology. His research interests are compound-protein interactions and bioinformatics.

**Xinyu Yu** is a master's student at Beijing University of Chemical Technology. Her research interests are variants calling and bioinformatics.

**Jingyang Gao** is a Professor at Beijing University of Chemical Technology. Her research interests include decoding Deep learning and bioinformatics.
