# Supplementary material for: Deletion variants calling in third-generation sequencing data based on a dual-attention mechanism
Source: Brief Bioinform. 2024 Jun 8;25(4):bbae269. doi: 10.1093/bib/bbae269 (PMC11162298; doi:10.1093/bib/bbae269)
Supplement: supplementary_bbae269 [file supplementary_bbae269.pdf]

Deletion variants calling in third-generation sequencing data based on a dual-attention mechanism

Running code for the tools

preparation work

minimap for genomic and spliced nucleotide sequences.

```
minimap2 -H -d ./data_chr4.mmi ./data_chr4.fa
minimap2 -t 8 -a
    --MD ./data_chr4.fa
    ./SRX1715702.fastq
    > ./SRX1715702.sam
samtools view -b -S ./SRX1715702.sam
    -o ./SRX1715702.bam
```

Add 'chr' to files.

```
samtools reheader
    - ./all_reads.fa.giab_h004_ngmlr-0.2.3_mapped.bam
    >  ./new-all_reads.fa.giab_h004_ngmlr-0.2.3_mapped.bam
```

Sort.

```
samtools sort
    -o  ./sort-data.bam
    -@ 20 ./data.bam
```

Index.

```
samtools index  ./sort-data.bam
```

Depth.

```
samtools depth ./sort-data.bam > ./sort-data.bam.txt
```

callers

cuteSV

cuteSV is a sensitive, fast, and scalable long-read-based structural variation (SV) detection approach. It uses tailored methods to comprehensively collect the signatures of various types of SVs, and a clustering-and-refinement method to analyze the signatures to implement a stepwise, highly sensitive SV detection. Benchmarks on simulated and real long-read sequencing datasets demonstrate that cuteSV has higher yields and scaling performance than state-of-the-art tools.

```
cuteSV
    --min_read_len 150
    --min_support 4
    --min_size 25
    ./sort-data.bam
```

```
    ./data.fa
    ./benchmark/cuteSV1.vcf
    ./benchmark/
```

SNIFFLES

SNIFFLES is a fast structural variant caller for long-read sequencing. It accurately detects structural variants (SVs) on germline, somatic, and population-level for PacBio and Oxford Nanopore read data. To call SVs from long read alignments (PacBio / ONT), Sniffles is used.

```
sniffles
    -s 3 -d 200  -t 4 -l 20 -r 100
    -m ./sort-data.bam
    -v ./sniffles.vcf
```

SVIM

SVIM, which stands for Structural Variant Identification Method, is a structural variant caller for third-generation sequencing reads. It is capable of detecting and classifying six classes of structural variation: deletions, insertions, inversions, tandem duplications, interspersed duplications, and translocations. Unlike other methods, SVIM integrates information from across the genome to precisely distinguish similar events, such as tandem and interspersed duplications and simple insertions.

```
svim alignment
    --min_sv_size 30
    --minimun_depth 3
    ./benchmark/
    ./sort-data.bam
    ./data.fa
```

PBSV

PBSV is a suite of tools to call and analyze structural variants in diploid genomes from PacBio single molecule real-time sequencing (SMRT) reads. It calls insertions, deletions, inversions, duplications, and translocations. Both single-sample calling and joint (multi-sample) calling are provided. PBSV is most effective for insertions 20 bp to 10 kb, deletions 20 bp to 100 kb, inversions 200 bp to 10 kb, duplications 20 bp to 10 kb, and translocations between different chromosomes or further than 100kb apart on a single chromosome.

```
pbsv discover
    -m 40 -y 10 -w 260 -a 25 -k 80
    ./data.bam
    ./data.bam.svsig.gz

pbsv call
    ./data.fa
    ./data.bam.svsig.gz
    ./pbsv.vcf
```

Combisv

```
perl combiSV2.0.pl
```

```
    —pbsv ./pbsv.vcf
    —sniffles ./sniffles.vcf
    —cutesv .v/cuteSV.vcf
    —svim ./svim.vcf
    —c 3 —o 1
```

simulate

Generate .fasta

```
./SURVIVOR simSV parameter_file
./SURVIVOR simSV ./chr2.fa parameter_file 0.1 0 ./simu_data
```

Install PaSS and run PaSS

```
PaSS.c
    —o PaSS
    —lm —lpthread
perl
    ./PaSS/pacbio_mkindex.pl ./simu_data.fasta ./simulation/
./PaSS
    —list percentage.txt
    —index index
    —m pacbio_RS
    —c ./sim.config
    —r 400000
    —t 16
    —o  ./chr2_simu_25X
```

Evaluation metrics

These metrics(Precision, Recall, F1-score) are usually used in binary classification problems, where the positive examples are the target categories we are interested in, and the negative examples are the categories other than the target categories.

Firstly, TP, FP, TN and FN are four important metrics used to evaluate the performance of classification models:

- TP (True Positive) is the number of positive samples that the model correctly predicts as positive.
- FP (False Positive) is the number of negative samples that the model incorrectly predicts as positive.
- TN (True Negative) is the number of negative samples that the model correctly predicts as negative.
- FN (False Negative) is the number of positive samples that the model incorrectly predicts as negative.

Precision

Precision is a measure of how many of the positive predictions made are correct (true positives). Formula:

$$Precision = \frac{TP}{TP + FP}$$

Recall

Recall is a measure of how many of the positive cases the classifier correctly predicted, over all the positive cases in the data. It is sometimes also referred to as Sensitivity. Formula:

$$Recall = \frac{TP}{TP + FN}$$

F1-Score

F1-Score is a measure combining both precision and recall. The F1 score is the reconciled average of Precision and Recall, which combines Precision and Recall, taking into account both the accuracy and the recall ability of the model. These metrics are commonly used to evaluate the performance of classification models in the presence of positive and negative sample imbalances, where Precision is concerned with the accuracy of the model and Recall is concerned with the comprehensiveness of the model, while F1-score combines both. Formula:

$$F1Score = \frac{2 * Precision * Recall}{Precision + Recall}$$

Machine learning terminology

One hot coding

If there are a total of N types of labels to construct one hot coding, a dictionary is constructed by using 0 $N-1$ integers corresponding to the elements one-to-one, and the i corresponding to each element is its index. To represent an element, a vector of length N is used, and the ith position of the vector is assigned as 1 and the rest of the positions are assigned as 0. The processed vector can then be input into the neural network for training.

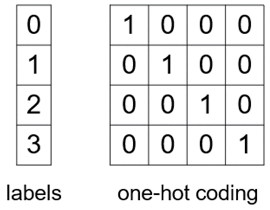

Fig. 1. Examples of one hot coding

Loss

Loss function is used to measure the difference between the model's predicted value $f(x)$ and the true value Y, it is a non-negative real-valued function, usually $L(Y, f(x))$ is used to represent, the smaller the loss function, the better the robustness of the model. The loss function is mainly used in the training phase of the model, after each batch of training data is fed into the model, the predicted value is output by forward propagation, and then the loss function will calculate the difference value between the predicted value and the real value, which is the loss value. After obtaining the loss value, the model updates each parameter by Back-propagation(BP) to reduce the loss between the true value and the predicted value, so that the predicted value generated by the model is closer to the true value, thus achieving the learning function.

Epoch and Batch

Epoch means that all the data is fed into the network, completing a forward calculation and a back propagation process. Since an epoch is often too large, it is divided into several small baches. It is not enough to iteratively train all the data once, it has to be repeated several times to fit and converge. In practice, all of the data is divided into several baches and fed a portion of the data at a time. As the number of epochs increases, the number of weight update iterations increases and the curve moves from the unfit state to the optimised fit state.

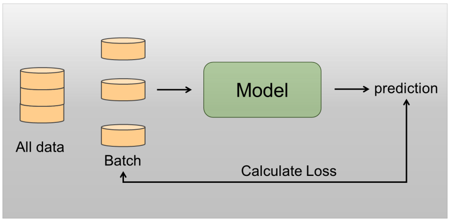

Fig. 2. One epoch. We will loop this process N times until the network converges.

CNN: convolutional neural networks

CNNs are a type of feed-forward neural network that learns feature engineering by itself via filters (or kernel) optimization. They are designed to automatically and adaptively learn spatial hierarchies of features from tasks with input data.

Convolutional Layer is the core building block of a CNN. The layer's parameters consist of a set of learnable filters (or kernels). These filters are small spatially (along width and height), but extend through the full depth of the input volume. During the forward pass, each filter is convolved across the width and height of the input volume, computing the dot product between the entries of the filter and the input and producing a 2-dimensional activation map of that filter. As a result, the network learns filters that activate when it detects some specific type of feature at some spatial position in the input.

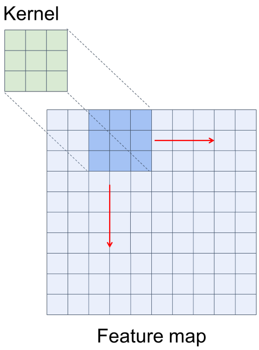

Fig. 3. Enter Caption

Global Average Pooling and Global max Pooling

Global Average Pooling (GAP): This operation calculates the average value of each feature map separately. It is designed to replace fully connected layers in classical CNNs. The idea is to generate a feature map for each corresponding category of the classification task in the last layer. Instead of adding fully connected layers on top of the feature maps, the average of each feature map is taken and the resulting vector is fed directly into the softmax layer. An advantage of GAP over the fully connected layers is that it is more native to the convolutional structure by enforcing correspondences between feature maps and categories. Another advantage is that there are no parameters to optimise in the GAP, thus avoiding overfitting in this layer. . Furthermore, GAP sums the spatial information and is therefore more robust to spatial translations of the input.
Global Max Pooling (GMP): This operation calculates the maximum value of each feature map over its entire spatial extent. It is similar to GAP, but instead of taking the average, it takes the maximum value of each feature map. GMP is commonly used to convert convolutional features of variable size images into a fixed size embedding. However, both GAP and GMP are computed spatially independent: each individual activation map is pooled, so activations from different locations are pooled together.

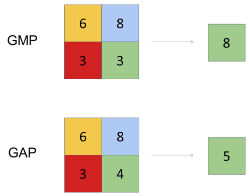

Fig. 4. Example of Global Average Pooling and Global max Pooling.

ResNet

The key feature of ResNet is the use of Residual Modules and Residual Connections to build the network, allowing deeper networks to be trained without the problem of gradient loss. Specifically, ResNet introduces the Shortcut Connection, which adds a cross-layer connection to each Residual Module, allowing information to be passed directly to later layers, preserving the original features and preventing them from disappearing layer by layer.

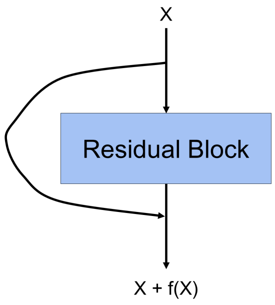

Fig. 5. Residual Block

LeakyReLU

LeakyReLU (Leakage Linear Rectifier Unit) is an activation function based on ReLU, but with a small slope in the negative part rather than a flat slope. This slope coefficient is determined before training, i.e. it is not learned during the training process.

$$LeakyReLU(x) = \begin{cases} \alpha x & \text{if } x < 0 \\ x & \text{if } x \geq 0 \end{cases}$$

Sigmoid

The sigmoid function is a type of mathematical function that has a characteristic S-shaped curve or sigmoid curve1. It is defined for all real input values and has a non-negative derivative at each point. It also has exactly one inflection point.

$$Sigmoid(x) = \frac{1}{1 + e^{-x}}$$

Batch Normalization

Batch normalisation, also known as batch norm, is a technique used to improve the training of deep neural networks. It was proposed in 2015 by Sergey Ioffe and Christian Szegedy.The main idea behind batch normalisation is to normalise the inputs of each layer so that they have a mean output activation of zero and a standard deviation of one. This is done for each mini-batch, hence the name ”batch normalisation”.

$$Input : B = \{x_{1...m}\}; \gamma, \beta(parameters\ to\ be\ learned) \tag{1}$$

$$Output : \{y_i = BN_{\gamma,\beta}(x_i)\} \tag{2}$$

$$\mu_B \leftarrow \frac{1}{m}\sum_{i=1}^{m} x_i \tag{3}$$

$$\sigma_B^2 \leftarrow \frac{1}{m}\sum_{i=1}^{m}(x_i - \mu_B)^2 \tag{4}$$

$$\tilde{x}_i \leftarrow \frac{x_i - \mu_B}{\sqrt{\sigma_B^2 + \epsilon}} \tag{5}$$

$$y_i \leftarrow \gamma \tilde{x}_i + \beta \tag{6}$$

$$\tag{7}$$

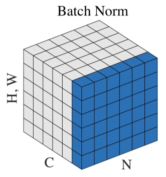

Fig. 6. Batch Normalization

Attention mechanism

The attention mechanism is a pivotal concept in the field of machine learning, particularly in the realm of natural language processing and neural networks. The Attention mechanism, if understood superficially, matches his name very well. His core logic is "From Attention to All to Attention to Focus". As a simple example, the following image has a dog in it, and we want to use a neural network to determine whether it is a dog or a cat. Normally, the background information of the image would affect the network's judgment, but the attention mechanism allows the algorithm to focus more on the area where the dog is located.

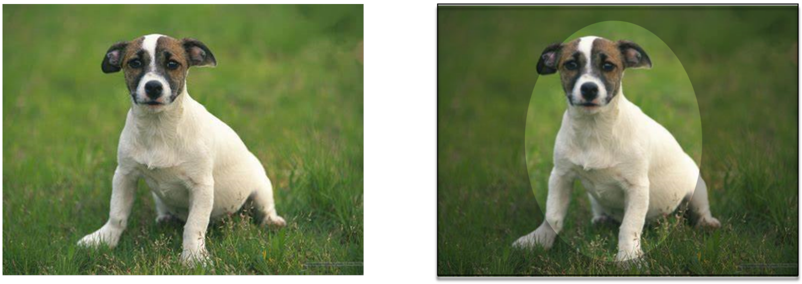

Fig. 7. Examples of Attention Mechanisms
